# A new species of *Thoracophelia* (Annelida, Opheliidae) from the Yellow Sea of South Korea

**DOI:** 10.3897/BDJ.12.e129526

**Published:** 2024-10-16

**Authors:** Ha-Eun Lee, Geon Hyeok Lee, Gi-Sik Min

**Affiliations:** 1 Department of Biological Sciences and Bioengineering, Inha University, Incheon, Republic of Korea Department of Biological Sciences and Bioengineering, Inha University Incheon Republic of Korea; 2 Research Institute of EcoScience, Ewha Womans University, Seoul, Republic of Korea Research Institute of EcoScience, Ewha Womans University Seoul Republic of Korea; 3 National Institute of Biological Resources, Incheon, Republic of Korea National Institute of Biological Resources Incheon Republic of Korea; 4 Department of Biological Sciences, Inha University, Incheon, Republic of Korea Department of Biological Sciences, Inha University Incheon Republic of Korea

**Keywords:** Opheliid, Polychaeta, taxonomy, DNA barcode

## Abstract

**Background:**

*Thoracophelia* Ehlers, 1897 is a genus of Opheliidae characterised by the body divided into three distinct regions, modified parapodia in chaetiger 10 and a ventral groove restricted to the posterior half of the body. To date, 18 species have been described in the genus. Amongst them, six species have been recorded in northeast Asia.

**New information:**

A new species, *Thoracopheliafoliformis* sp. nov., was discovered in the intertidal zone of the Yellow Sea, South Korea. This is the first *Thoracophelia* species report from the Yellow Sea. This new species is closely related to *T.dillonensis* (Hartman, 1938) from California and *T.ezoensis* Okuda, 1936 from Japan in having pectinate branchiae. However, the new species can be distinguished from the two species by the unique combination of the following characteristics: 15 pairs of wrinkled pectinate branchiae with 12–15 filaments at best development and a foliaceous mid-ventral plate in the pygidium instead of one or two thick ventral cirri. Detailed descriptions and illustrations of *T.foliformis* sp. nov. are provided. Sequences of the mitochondrial cytochrome c oxidase subunit I (COI), nuclear 18S ribosomal DNA (rDNA) and 28S rDNA of the new species were determined and analysed.

## Introduction

Species in the family Opheliidae Malmgren, 1867 are widely distributed worldwide, from shallow to deep seas and are mainly found in sandy sediments (Parapar et al. 2021). Opheliids are burrowing, deposit-feeding polychaetes characterised by a pointed prostomium, smooth cuticle and conspicuous ventral groove (Blake 2000). Opheliidae currently comprises 172 species belonging to eight genera (Read and Fauchald 2024).

The genus *Thoracophelia*, erected by Ehlers (1897) with the type species *Thoracopheliafurcifera*, is characterised by having modified parapodia in chaetiger 10, a ventral groove restricted to the posterior half of the body and three distinct body regions: the cephalic, thoracic and abdominal regions (Law et al. 2013, Parapar et al. 2021). Whereas some opheliids with those morphological features were previously regarded as members of the genera *Euzonus* Grube, 1866, *Pectinophelia* Hartman, 1938 and *Lobochesis* Hutchings & Murray, 1984 (Grube 1866, Hartman 1938, Hartman 1956, Hutchings and Murray 1984, Brewer et al. 2011), those genera were later synonymised with the genus *Thoracophelia* (Santos et al. 2004, Blake 2011). Thus, *Thoracophelia* is the only valid genus for the opheliids having three body regions and comprises 18 species (Read and Fauchald 2024).

To date, six species have been recorded in Northeast Asia: *Thoracopheliaarctica* (Grube, 1866), *Thoracopheliaezoensis* Okuda, 1936, *Thoracopheliajaponica* (Misaka & Sato, 2003), *Thoracopheliaminuta* Jimi et al., 2021, *Thoracopheliawilliamsi* (Hartman, 1938) and *Thoracopheliayasudai* Okuda, 1934 (Song et al. 2017, Jimi et al. 2021). Amongst those species, one species, *T.williamsi*, has been reported in South Korea (Song et al. 2017).

In the present study, we discovered a new *Thoracophelia* species in the intertidal zone of the Yellow Sea. Detailed descriptions and illustrations of *T.foliformis* sp. nov. have been provided. Sequences of the mitochondrial cytochrome c oxidase subunit I (COI), nuclear 18S ribosomal DNA (rDNA) and 28S rDNA were determined for DNA barcoding and phylogenetic studies.

## Materials and methods

### Sampling and morphological observations

**Figure 1.**
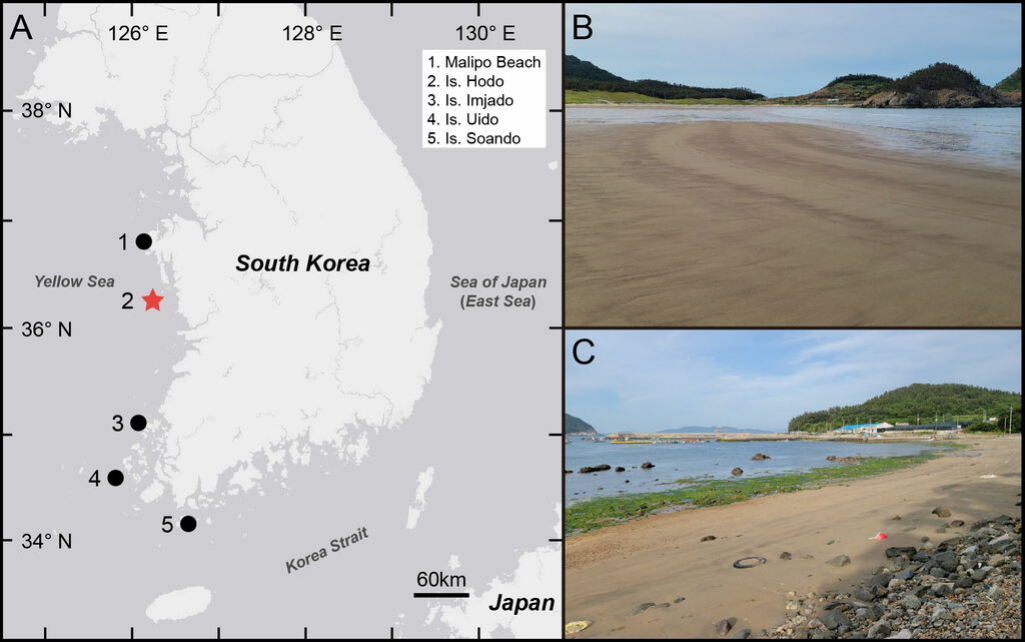
Collection sites of *Thoracopheliafoliformis* sp. nov. **A** map of collection sites: type locality (red star) and other collection sites (black circle); **B, C** general view of habitat.

Specimens were collected from the upper intertidal zone of the Yellow Sea, South Korea during March 2021 and June 2022 (Fig. 1). The sediment was carefully collected using a scoop and gently sieved with seawater to collect the polychaete samples. Live specimens were relaxed by using 10% magnesium chloride (MgCl_2_) solution and morphological characteristics were observed under a stereoscopic microscope (SZX12; Olympus, Japan). Samples for DNA extraction were fixed in 95% ethanol, whereas the other samples were fixed in 4% formaldehyde solution and preserved in 95% ethanol. Additional morphological observations were conducted under a stereoscopic microscope and photographs of the specimens were captured using a digital camera (Dhyana 400DC; Tucsen, China) with the Mosaic capture programme (Mosaic version 15; Tucsen, China). Some specimens were stained with methyl green solution to investigate the staining pattern. Specimens for scanning electron microscopy (SEM) were dried using a critical point dryer (E3000; Polaron, UK), covered with platinum and observed using a Hitachi S-4300SE (Hitachi, Japan). Voucher specimens were deposited at the National Institute of Biological Resources (NIBR) in Incheon, South Korea.

### Molecular analysis

DNA was extracted from three ethanol-preserved specimens (NIBRIV0000900977, NIBRIV0000910953 and NIBRIV0000910956) using LaboPass Tissue Mini (Cosmo GENETECH, South Korea) according to the manufacturer’s instructions. The partial sequences of COI, 18S rDNA and 28S rDNA were amplified using the following primer sets: PolyLCO/PolyHCO (Carr et al. 2011) for COI, 1F/5R, 3F/BI and A2.0/9R (Giribet et al. 1996, Whiting et al. 1997, Giribet et al. 1999) for 18S rDNA and Po28F1/Po28R4 (Struck et al. 2006) for 28S rDNA. The obtained COI, 18S rDNA and 28S rDNA sequences were edited and aligned using Geneious 8.1.9 (Biomatters, New Zealand). Intra- and interspecific genetic distances were calculated by p-distances using Geneious 8.1.9. Newly-determined DNA sequences were registered in GenBank (PP903708–10 for COI, PP905510–12 for 18S rDNA and PP905507–9 for 28S rDNA). Maximum Likelihood (ML) and Bayesian Inference (BI) analyses were performed on the three genes. Sequences of *Ophelialimacina* (Rathke, 1843) and *Polygordiuslacteus* Schneider, 1868 were used as outgroup taxa. Maximum Likelihood (ML) analyses were conducted in IQ-TREE v.1.6.1 using the GTR+F+I, K2P+I, TIM3+F+G4 and TN+F+R2 models for the three genes and the concatenated nuclear genes, respectively, with 1,000 replicates (Kalyaanamoorthy et al. 2017). Bayesian Inference (BI) analyses were performed in MrBayes v.3.2.6 (Ronquist and Huelsenbeck 2003) with the TIM3+G, TrN+I+G, TIM3+I+G and TIM2+I+G models selected by the Akaike Information Criterion (AIC) implemented in jModelTest v.2.1.10 (Darriba et al. 2012).

## Taxon treatments

### Thoracophelia

Ehlers, 1897

7B31DFDA-2919-5231-9419-4FABD19EF754

#### New Korean generic name

Keun-yo-jeong-get-ji-reong-yi (큰요정갯지렁이속)

### Thoracophelia foliformis

Lee, Lee & Min, 2024 sp. nov.

37144B49-821C-51FB-97A0-E974F0E0D710

4E6F2CED-9140-43F5-BF06-3578DEC7FABA

#### Materials

- **Type status:** Holotype. **Occurrence:** catalogNumber: NIBRIV0000900976; individualCount: 1; lifeStage: adult; occurrenceID: FF9733A0-0010-58EA-BB06-AD28627CAD15; **Taxon:** scientificName: *Thoracopheliafoliformis*; phylum: Annelida; class: Polychaeta; family: Opheliidae; genus: Thoracophelia; specificEpithet: *foliformis*; scientificNameAuthorship: Lee, Lee & Min; **Location:** country: South Korea; municipality: Boryeong-si; locality: Is. Hodo; verbatimLatitude: 36°17’54” N; verbatimLongitude: 126°15’58” E; **Event:** eventDate: 08-09-2021
- **Type status:** Paratype. **Occurrence:** catalogNumber: NIBRIV0000900977, 0910950–910952; individualCount: 18; lifeStage: adult; occurrenceID: 30FB9856-7E6A-571C-8474-A177E17B3759; **Taxon:** scientificName: *Thoracopheliafoliformis*; phylum: Annelida; class: Polychaeta; family: Opheliidae; genus: Thoracophelia; specificEpithet: *foliformis*; scientificNameAuthorship: Lee, Lee & Min; **Location:** country: South Korea; municipality: Boryeong-si; locality: Is. Hodo; verbatimLatitude: 36°17’54” N; verbatimLongitude: 126°15’58” E; **Event:** eventDate: 08-09-2021
- **Type status:** Paratype. **Occurrence:** catalogNumber: NIBRIV0000910953; individualCount: 1; lifeStage: adult; occurrenceID: DEF1D918-59D7-5AC8-9BC3-C86B1BFC2C00; **Taxon:** scientificName: *Thoracopheliafoliformis*; phylum: Annelida; class: Polychaeta; family: Opheliidae; genus: Thoracophelia; specificEpithet: *foliformis*; **Location:** country: South Korea; municipality: Taean-gun; locality: Malipo Beach; verbatimLatitude: 36°47’16” N; verbatimLongitude: 126°08’27” E; **Event:** eventDate: 29-03-2021; **Record Level:** institutionCode: NIBR
- **Type status:** Paratype. **Occurrence:** catalogNumber: NIBRIV0000910954; individualCount: 1; lifeStage: adult; occurrenceID: 15E441E9-2197-5DB6-BA13-F74C062A5BA3; **Taxon:** scientificName: *Thoracopheliafoliformis*; phylum: Annelida; class: Polychaeta; family: Opheliidae; genus: Thoracophelia; specificEpithet: *foliformis*; scientificNameAuthorship: Lee, Lee & Min; **Location:** country: South Korea; municipality: Wando-gun; locality: Is. Soando; verbatimLatitude: 34°09’56” N; verbatimLongitude: 126°39’28” E; **Event:** eventDate: 25-05-2021
- **Type status:** Paratype. **Occurrence:** catalogNumber: NIBRIV0000910955; individualCount: 1; lifeStage: adult; occurrenceID: 2F73D89A-D734-57BA-80F8-444D54780807; **Taxon:** scientificName: *Thoracopheliafoliformis*; phylum: Annelida; class: Polychaeta; family: Opheliidae; genus: Thoracophelia; specificEpithet: *foliformis*; scientificNameAuthorship: Lee, Lee & Min; **Location:** country: South Korea; municipality: Sinan-gun; locality: Is. Imjado; verbatimLatitude: 35°06’18” N; verbatimLongitude: 126°04’10” E; **Event:** eventDate: 22-07-2021
- **Type status:** Paratype. **Occurrence:** catalogNumber: NIBRIV0000910956–910958; individualCount: 3; lifeStage: adult; occurrenceID: 30EFEDB9-3B6B-5AF9-BC6E-B9DFDAB7C843; **Taxon:** scientificName: *Thoracopheliafoliformis*; phylum: Annelida; class: Polychaeta; family: Opheliidae; genus: Thoracophelia; specificEpithet: *foliformis*; scientificNameAuthorship: Lee, Lee & Min; **Location:** country: South Korea; municipality: Sinan-gun; locality: Is. Uido; verbatimLatitude: 34°36’26” N; verbatimLongitude: 125°49’38” E; **Event:** eventDate: 01-06-2022

#### Description

Holotype complete with 38 chaetigers, 17.2 mm long, 1.6 mm wide in thoracic region and 1.3 mm wide in abdominal region. Paratypes with 37–38 chaetigers, 11.1–24.2 mm long, 1.3–2.0 mm wide. Live specimens reddish in colour and body wall transparent. The reddish colour gradually fades to yellow or cream after alcohol preservation, but can remain for a year in the case of formalin fixation.

**Figure 2.**
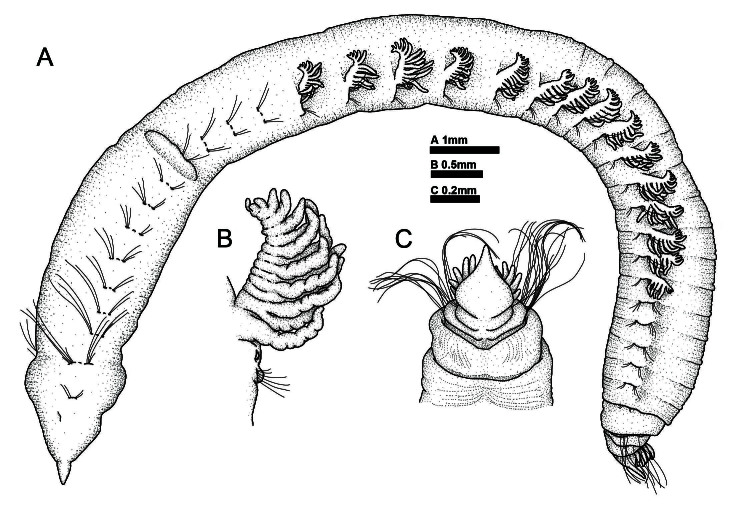
*Thoracopheliafoliformis* sp. nov. **A, B** holotype (NIBRIV0000900976); **C** paratype (NIBRIV0000910950). **A** whole body, lateral view; **B** branchiae; **C** pygidium, ventral view. Scale bars: A = 1 mm; B = 0.5 mm; C = 0.2 mm.

**Figure 3.**
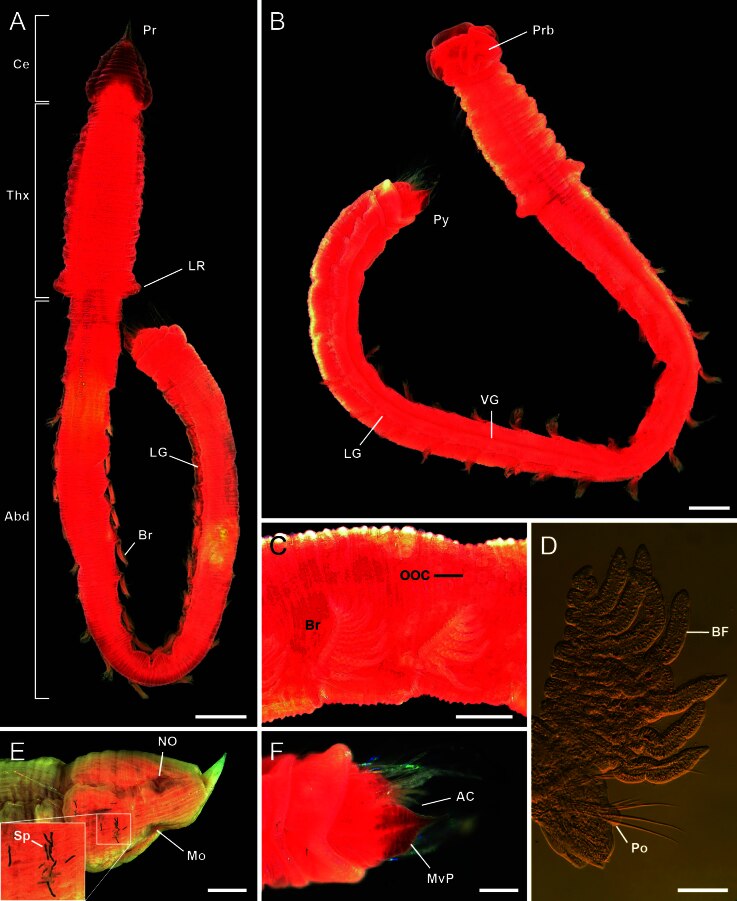
Photographs of *Thoracopheliafoliformis* sp. nov. **A** paratype (NIBRIV0000910958); **B, F** paratype (NIBRIV0000910957); **C, E** paratype (NIBRIV0000910956). **A–C, F** live specimens. **A** whole body, dorsal view; **B** whole body with everted proboscis, ventral view; **C** abdominal segments, lateral view; **D** branchiate parapodia; **E** cephalic region, lateral view; **F** mid-ventral plate of pygidium, ventral view. Abbreviation: Abd = abdomen, AC = anal cirrus, BF = branchial filament, Br = branchiae, Ce = cephalic region, LG = lateral groove, LR = lateral ridge, Mo = mouth, MvP = mid-ventral plate, NO =nuchal organ, OOC = oocyte, Po = postchaetal lobe, Pr = prostomium, Prb = proboscis, Py = pygidium, Sp = spicule, Thx = thorax, VG = ventral groove. Scale bars: A, B = 1 mm; C, E, F = 0.5 mm; D = 100 µm.

**Figure 4.**
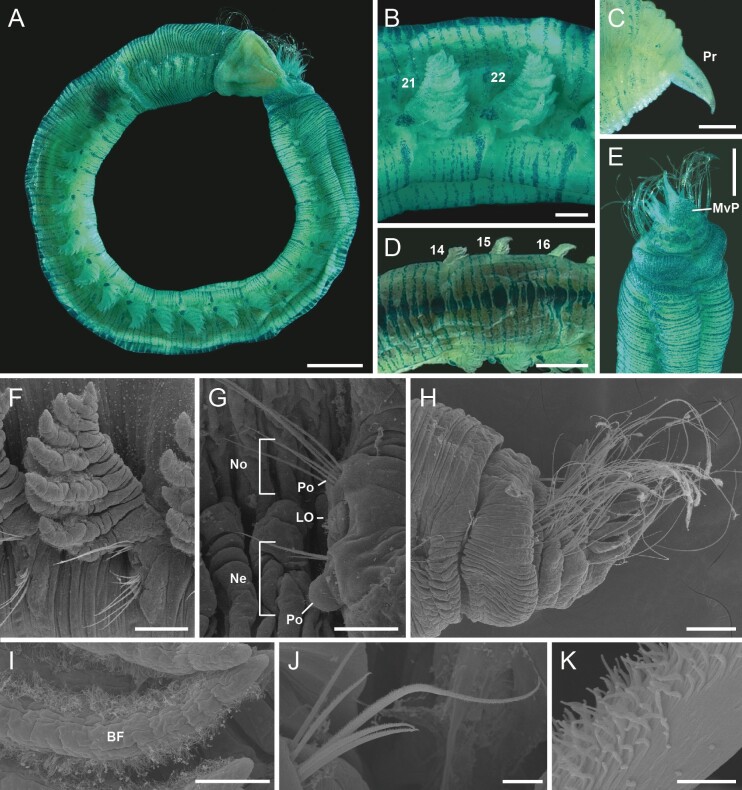
Staining patterns and SEM images of *Thoracopheliafoliformis* sp. nov. **A–E** paratype (NIBRIV0000910952); **F, G** paratype (NIBRIV0000910951); **H–K** paratype (NIBRIV0000910954). **A** whole body, lateral view; **B** circular patch at the base of branchiae; **C** prostomium; **D** dorsal pattern; **E** pygidium; **F** branchiae; **G** parapodia, anterior view; **H** pygidium, lateral view; **I** branchial filament; **J** serrated chaetae; **K** serration of chaeta. Abbreviation: LO = lateral organ, Ne = neuropodia, No = notopodia. Scale bars: A = 1 mm; B, C, H = 0.2 mm; D, E = 0.5 mm; F = 100 µm; G, I = 50 µm; J = 10 µm; K = 1 µm.

Body distinctly divided into three regions by weak constrictions; cephalic, thoracic, and abdominal regions (Fig. 2A and Fig. 3A). The cephalic region consisting of a pointed prostomium and two setigers. A pair of nuchal organs present in cephalic region dorsolaterally (Fig. 2A and Fig. 3E). Multilobulated proboscis eversible from mouth region (Fig. 3B and E). The thoracic region inflated and consisting of eight chaetigers (Fig. 3A and B). Each chaetiger with about five indistinct annulations. Chaetiger 10 with smooth lateral ridge (Fig. 2A and Fig. 3A). The abdominal region includes 17–18 chaetigers and pygidium (Fig. 3A). Last 3–4 chaetigers decreasing in size (Fig. 2A, Fig. 3B and Fig. 4H). Longitudinal mid-ventral groove extending from about chaetiger 10 to pygidium (Fig. 3B). A pair of longitudinal lateral grooves present in the abdominal region, one on each side of body (Fig. 3A and B). Body formula 13a (abranchiate) + 15b (branchiate) + 9–10a (posterior abranchiate) = 37–38 chaetigers. Black rod-shaped spicules in coelom cavity corrugate or curve, with about 38–98 µm in length (Fig. 3E).

Branchiae pectinate, wrinkled, occurring from chaetigers 14 to 28 (Fig. 2B, Fig. 3C, D and Fig. 4B and F). Branchiae with finger-shaped branchial filaments from the main stem; the well-developed ones bear up to 12–15 filaments. Each branchial filaments with cilia on dorsal and ventral side (Fig. 4Fand I).

Parapodia biramous, with rounded postchaetal lobes on each noto- and neuropodia (Fig. 4G). Notopodial postchaetal lobes lower than neuropodial ones. Lateral organs present in between noto- and neuropodia, with a longitudinally arranged row of cilia (Fig. 4G).

Chaetae distally serrated in one side (Fig. 4J and K). Chaetae distinctly longer in chaetigers 3–5 and posterior 3–4 chaetigers (Fig. 2A).

Pygidium with about 6–8 anal cirri on each lateral side and a foliaceous mid-ventral plate, flattened, wider basally and distally tapering (Fig. 2C, Fig. 3F and Fig. 4H).

#### Diagnosis

Fifteen pairs of branchiae present on chaetiger 14–28. Branchiae pectinate, wrinkled and the well-developed ones bear 12–15 finger-shaped branchial filaments. Chaetiger 10 with a pair of smooth lateral ridge. Pygidium with a foliaceous mid-ventral plate and about 6–8 anal cirri on each lateral side.

#### Etymology

The new specific name derives from the foliaceous shape of the mid-ventral plate in the pygidium. The name is a combination of the Latin words *folium* (meaning ‘leaf’) and *formis* (meaning ‘shape’).

#### Distribution

The new species was collected from the upper intertidal zone, which consists of sand or muddy sand, in the Yellow Sea of South Korea (Fig. 1).

#### Variation

Although the number of branchial filaments in well-developed branchiae is variable (12–15), there was no variation in the number of pairs of branchiae (15 pairs) or the first branchiae-bearing chaetiger (chaetiger 14) amongst the specimens.

#### Reproductive information

Female adult specimens were observed in the present study. Oocytes present in coelom cavity, with about 0.1 mm diameter, visible through the transparent body wall (Fig. 3C).

#### Methyl green staining pattern (MGSP)

Annulations of each segment have weak line-shaped stain and the stains are thicker at mid-dorsal abdominal region (Fig. 4A, B and D). Several posteriormost segments and the mid-ventral plate of pygidium are intensely stained (Fig. 4E). A deep and large circular patch present at the base of each branchiae anteriorly (Fig. 4A and B). Prostomium with weak stain on lateral sides (Fig. 4C).

#### Genetics

Sequences of mitochondrial COI, nuclear 18S rDNA and 28S rDNA were obtained from the three specimens of *Thoracopheliafoliformis* sp. nov. The intraspecific variation in 28S rDNA (849 bp) was 0.1%; however, there was no intraspecific variation in COI (657 bp) or 18S rDNA (1,684 bp). The interspecific variations between the new species and the congeners were 14.2–15.4% in COI (654 bp), 0.2–0.3% in 18S rDNA (1,750 bp) and 3.6–4.2% in 28S rDNA (873 bp) (Table 1).

#### Remarks

*Thoracopheliafoliformis* sp. nov. is the first *Thoracophelia* species described in the Yellow Sea. In possessing pectinate branchiae, *Thoracopheliafoliformis* sp. nov. resembles *Thoracopheliadillonensis* (Hartman, 1938) from California and *Thoracopheliaezoensis* Okuda, 1936 from Japan. While most similar to *T.dillonensis* in having 15 pairs of branchiae, the new species is distinguished from *T.dillonensis* by the shape of its branchiae and pygidium. The new species has wrinkled branchiae with 12–15 filaments at best development and a foliaceous mid-ventral plate in the pygidium, whereas *T.dillonensis* has unwrinkled, smooth branchiae with 15–20 filaments at best development and two thick mid-ventral cirri instead of the plate. *Thoracopheliaezoensis* also differs from the new species as the former possesses 19 pairs of branchiae and a thick mid-ventral cirrus, whereas the latter has 15 pairs of branchiae and a foliaceous mid-ventral plate. Furthermore, the new species also differs from the two species, as the branchiae of the new species first appear on chaetiger 14, while those of the two species first appear on chaetiger 13. In Northeast Asia, six species have been reported and are clearly distinguished from the new species by the shape of the branchiae, except *T.ezoensis* mentioned above. Morphological differences amongst those species are mentioned in the identification keys below.

#### New Korean name

Yip-sa-gwi-Keun-yo-jeong-get-ji-reong-yi (잎사귀큰요정갯지렁이)

## Identification Keys

### Key to *Thoracophelia* species in Northeast Asia

**Table:** 

1	Branchiae with 3 or less branchial filaments	2
–	Branchiae with more than 3 branchial filaments	3
2	Branchiae simple, hemisphere-shaped	*T.minuta* Jimi et al., 2021
–	Branchiae bifid and sometimes trifid	*T.williamsi* (Hartman, 1938)
3	Branchiae dichotomously branched	4
–	Branchiae not dichotomously branched	5
4	With 15 pairs of branchiae	*T.yasudai* Okuda, 1934
–	With 17 pairs of branchiae	*T.arctica* (Grube, 1866)
5	Branchiae palmatifid; Chaetiger 10 with 11 dorsal and 3 ventral conical cirri	*T.japonica* (Misaka & Sato, 2003)
–	Branchiae pectinate; Chaetiger 10 with smooth lateral ridge	6
6	With 19 pairs of branchiae, first appearing on chaetiger 13	*T.ezoensis* Okuda, 1936
–	With 15 pairs of branchiae, first appearing on chaetiger 14	***T.foliformis* sp. nov.**

## Discussion

**Figure 5.**
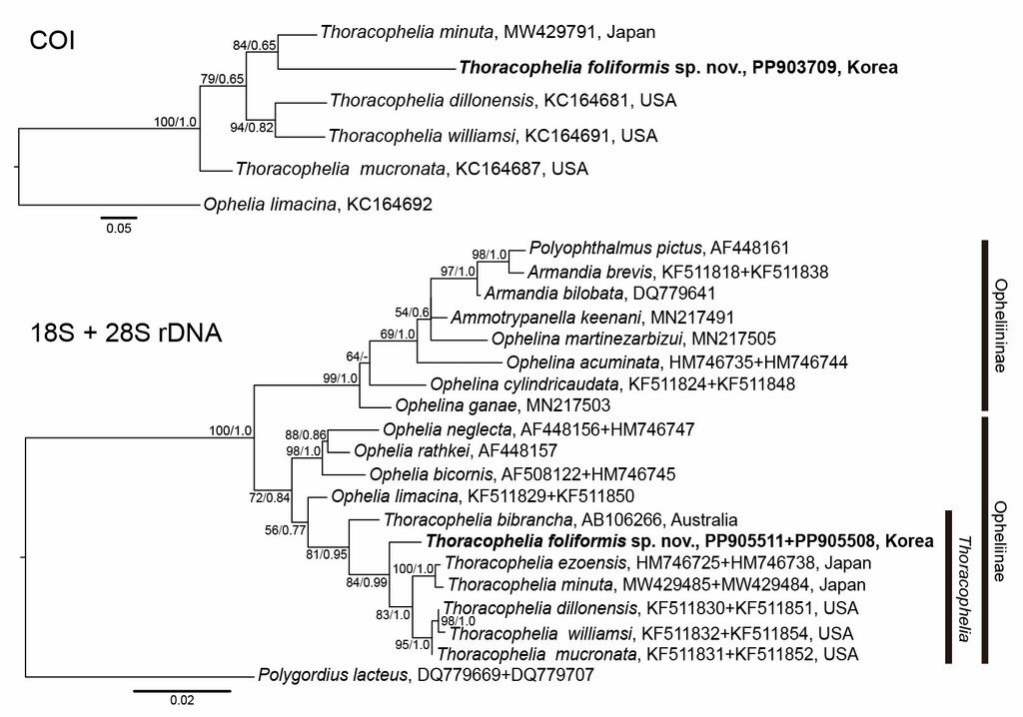
Maximum Likelihood (ML) and Bayesian Inference (BI) analyses, based on COI and 18S+28S rDNA sequences. The numbers at nodes represent the ML bootstrap values of ≥ 50% and the BI posterior probabilities of ≥ 0.5. New species is in bold. *Ophelialimacina* and *Polygordiuslacteus* were used as outgroup taxa.

To date, 18 species have been described in the genus *Thoracophelia*. The new species is clearly distinguished from the previously-described *Thoracophelia* species, based on the shape of the branchiae and pygidium. In possessing pectinate branchiae, *T.foliformis* sp. nov. resembles *T.dillonensis* and *T.ezoensis*. However, the new species is distinguished from *T.dillonensis* by its wrinkled branchiae with fewer branchial filaments (12–15 vs. 15–20) at full development and a mid-ventral plate in the pygidium. Although the number of branchial filaments is a variable characteristic defined in the range, a 15.4% dissimilarity in COI sequences indicates that the new species is clearly distinguished from *T.dillonensis* (Table 1, Fig. 5). The new species differs from *T.ezoensis* in the number of pairs of branchiae (15 vs. 19) and the presence of a mid-ventral plate in the pygidium. The new species also differs from *T.minuta* Jimi et al., 2021, which has a minute body and simple hemisphere-shaped branchiae without a ciliated area. The new species is distinguished from *T.japonica* as the latter possesses palmatifid branchiae with 3–7 filaments and a pair of lateral transverse rows of conical cirri on chaetiger 10 instead of a lateral ridge (Misaka and Sato 2003). The new species is also distinguished from *T.yasudai* and *T.arctica*, as the two species possess dichotomously divided branches (Grube 1866, Okuda 1934). By possessing bifid or trifid branchiae, the other twelve *Thoracophelia* species are also distinguished from the new species (Santos et al. 2004).

In Korea, *T.williamsi* has been reported from Sea of Japan without description and deposition information by Song et al. (2017). The new species described in this paper is clearly distinguished from *T.williamsi* because the former has pectinate branchiae, while the latter possess bifid and trifid branchiae with pinnates on dorsal filaments (Hartman 1938).

Recent studies on Opheliidae using scanning electron microscopy (SEM) have revealed the presence of ciliated areas on lateral organs and branchiae and the diversity in the structure of lateral and nuchal organs, which may be reliable taxonomic characters for future phylogenetic studies (Parapar et al. 2011, Parapar et al. 2021). In this study, the presence of ciliated areas in the lateral organs and the branchial filament was first observed and described in the genus *Thoracophelia*, whereas these characteristics are not discernible in the SEM images of *T.minuta*. Thus, these characteristics could be useful morphological features if investigated in other *Thoracophelia* species.

Staining methods have been used for species identification in several annelid taxa by observing indistinct surface features or revealing specific staining patterns (Winsnes 1985, Kongsrud et al. 2011, Blake 2019). In the present study, methyl green staining patterns (MGSP) on *T.foliformis* sp. nov. were observed and described. As MGSPs have not been examined in other Thoracophelia species, it is necessary to further investigate the staining pattern in more species in order to evaluate whether the observed MGSPs have specific or generic features.

In COI, the minimum interspecific variation between the new species and congeners was 14.2% for *T.minuta* (Table 1, Fig. 5). However, the new species differs from *T.minuta* in the shape of its branchiae (pectinate vs. hemispherical). Although the new species is most closely related to *T.mucronata* with 0.2% and 3.6% dissimilarities in 18S and 28S rDNA (Table 1), respectively, the new species differs from *T.mucronata* by having pectinate branchiae instead of bifid ones. In the tree based on the concatenated 18S and 28S rDNA sequences, Opheliidae was clustered into two clades of subfamilies Ophelininae and Opheliinae, as in previous studies, based on the concatenated sequences of several gene regions (Paul et al. 2010, Law et al. 2014, Jimi et al. 2021) (Fig. 5). *Thoracophelia* species formed a monophyletic group, including the new species, with high support values (ML = 81%, BI = 0.95) (Fig. 5). The new species is sister to the two clades of Japanese and American *Thoracophelia* species, although the morphological features reflecting these phylogenetic relationships have not yet been identified (Fig. 5). Trees of 18S and 28S rDNA sequences showed results similar to those of the concatenated nuclear genes and are provided as a supplementary file (Suppl. material 1).

## Supplementary Material

XML Treatment for Thoracophelia

XML Treatment for Thoracophelia foliformis

89EF96A3-E60D-523B-94E9-8D95FFB055F6 10.3897/BDJ.12.e129526.suppl1 Supplementary material 1 Trees of 18S and 28S rDNA sequences using Maximum Likelihood (ML) and Bayesian Inference (BI) analyses Data type Figure Brief description Maximum Likelihood (ML) and Bayesian Inference (BI) analyses, based on 18S and 28S rDNA sequences. The numbers at nodes represent the ML bootstrap values of ≥ 50% and the BI posterior probabilities of ≥ 0.5. New species is in bold. *Polygordiuslacteus* was used as an outgroup taxon. File: oo_1127042.docx https://binary.pensoft.net/file/1127042 Ha-Eun Lee

## Figures and Tables

**Table 1.** Interspecific genetic distances of three genes (COI, 18S rDNA and 28S rDNA) amongst *Thoracophelia* species and an outgroup species, *Ophelialimacina*.

Species		Interspecific genetic distance						GenBank accession No.
COI (654 bp aligned)		1	2	3	4	5		
1	*T.foliformis* sp. nov.							PP903709
2	*T.dillonensis*	0.154						KC164681
3	*T.minuta*	0.142	0.115					MW429791
4	*T.mucronata*	0.147	0.126	0.122				KC164687
5	*T.williamsi*	0.146	0.091	0.116	0.116			KC164691
6	*Ophelialimacina*	0.175	0.187	0.185	0.169	0.176		KC164692
18S rDNA (1,750 bp aligned)		1	2	3	4	5	6	
1	*T.foliformis* sp. nov.							PP905511
2	*T.dillonensis*	0.003						KF511830
3	*T.ezoensis*	0.003	0.003					HM746725
4	*T.minuta*	0.003	0.003	0.001				MW429485
5	*T.mucronata*	0.002	0.001	0.002	0.001			KF511831
6	*T.williamsi*	0.003	0.001	0.003	0.003	0.002		KF511832
7	*Ophelialimacina*	0.006	0.007	0.005	0.004	0.006	0.006	KF511829
28S rDNA (873 bp aligned)		1	2	3	4	5	6	
1	*T.foliformis* sp. nov.							PP905508
2	*T.dillonensis*	0.037						KF511851
3	*T.ezoensis*	0.042	0.025					HM746738
4	*T.minuta*	0.039	0.026	0.007				MW429484
5	*T.mucronata*	0.036	0.001	0.024	0.024			KF511852
6	*T.williamsi*	0.042	0.005	0.031	0.031	0.008		KF511854
7	*Ophelialimacina*	0.067	0.066	0.075	0.077	0.067	0.066	KF511850
