# Supplementary material for: A new species of Thoracophelia (Annelida, Opheliidae) from the Yellow Sea of South Korea
Source: Biodivers Data J. 2024 Oct 16;12:e129526. doi: 10.3897/BDJ.12.e129526 (PMC11499668; doi:10.3897/BDJ.12.e129526)
Supplement: Supplementary material 1 — Trees of 18S and 28S rDNA sequences using Maximum Likelihood (ML) and Bayesian Inference (BI) analyses [file bdj-12-e129526-s001.docx]

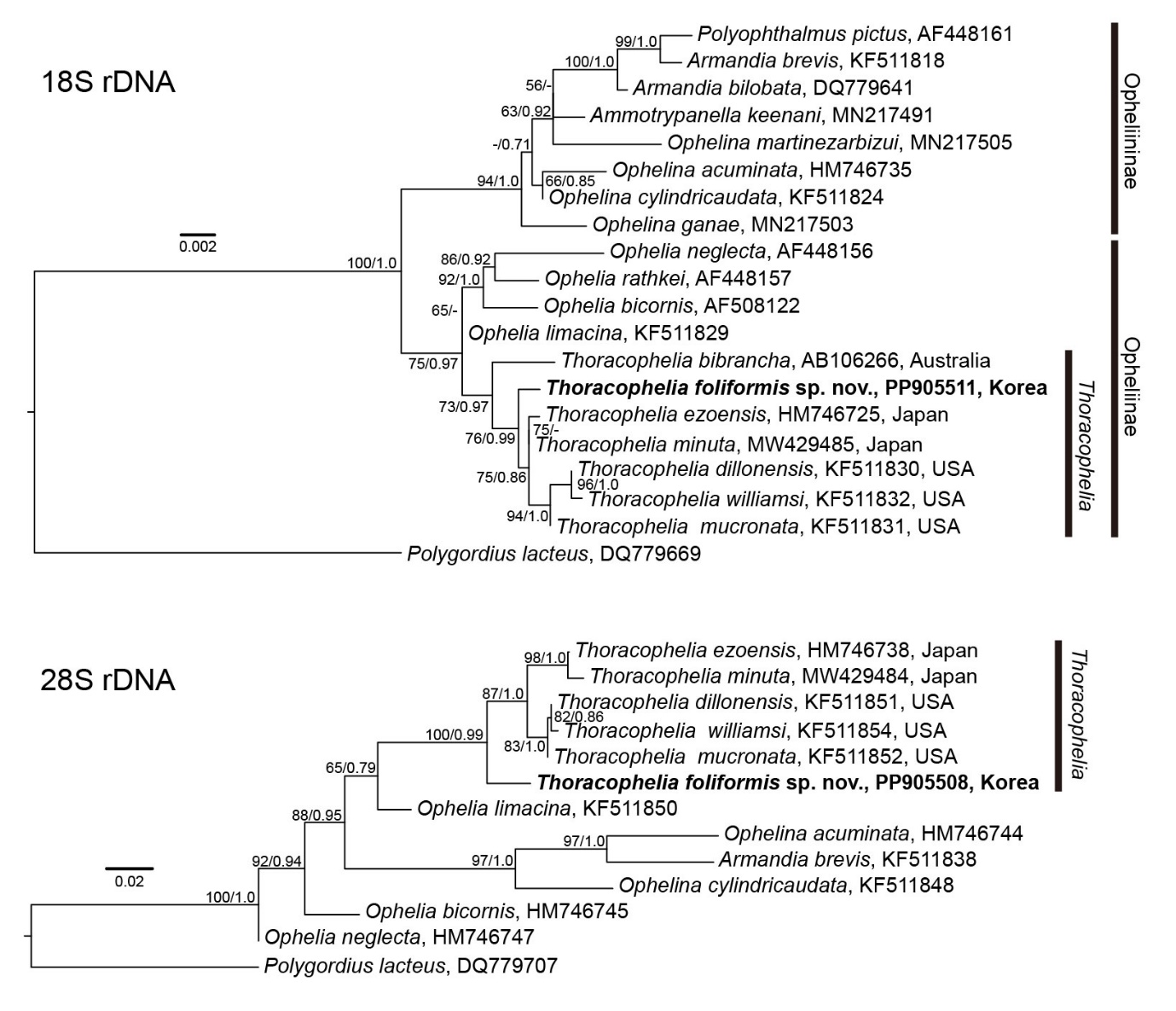


**Supp. Material 1.** Maximum Likelihood (ML) and Bayesian Inference (BI) analyses, based on 18S and 28S rDNA sequences. The numbers at nodes represent the ML bootstrap values of ≥ 50% and the BI posterior probabilities of ≥ 0.5. New species is in bold. *Polygordius lacteus* was used as an outgroup taxon.
